# Report of a Case of Intractable Vomiting of Pregnancy Treated by Electric Currents and Illustrating the Importance of Current Differentiation*Read before Orleans Parish (Louisiana) Medical Society.

**Published:** 1904-06

**Authors:** Amedee Granger

**Affiliations:** New Orleans, La., Fellow American Electro-Therapeutic Association


For Texas Medical Journal.

### Report of a Case of Intractable Vomiting of Pregnancy Treated by Electric Currents and Illustrating the Importance of Current Differentiation.*

BY AMEDEE GRANGER, M. D., NEW ORLEANS, LA.,
Fellow American Electro-Therapeutic Association.

To correctly differentiate between the various electric modalities, and select the one best suited to the treatment of a diseased condition, is the most important and at the same time the most neglected part of the art of electro-therapeutics. It is true that the laity and, I am sorry to add, the majority of our own profession, regard the different electric currents as one remedy about which as yet little is known. Nothing is more false, for although we know as yet nothing of the exact nature of electricity, we understand the laws governing the electrical forces, and the mechanical, chemical, physical, and physiological effects of the various manifestations of electricity have been carefully studied and recorded.

To the electro-therapeutist electricity therefore becomes an agent which by being variously modified in amperage and voltage, or by being interrupted becomes capable of as many distinct and varied mechanical, physical, chemical and physiological effects, just as so many different drugs.

For example, although all the electric modalities have nutritional effects, due to the influence of electricity on the circulation, secretion, excretion and absorption, it is conceded by the best authorities that to increase nutritional activity and the general metabolic processes of the body, the currents of high potential and high frequency—static and high frequency—are pre-eminent.

On the other hand, Graham's currents, or to be more correct, continuous currents, owing to their large amperage alone possess the chemical or electro-lytic and cataphoric effects. These are very important when we remember that upon them depend the contracting, astringent, hemorrhagic, antiseptic, germicidal and caustic actions of the positive pole; and the dilating, nutritional, relaxing, hemorrhagic and escharotic action of the negative pole.

Now for mechanical effects, we select the currents of alternations—Faradic and Sinusoidal. The Faradic from short and coarse wire for muscular contraction, and from long fine wire for

*Read before Orleans Parish (Louisiana) Medical Society.

vaso-motor effects. The later produce sedation or stimulation, and local or reflex vaso-motor dilatation or contraction, according to the technique employed and length of wire used.

My object in presenting the report of this case is four-fold:

First. As an illustration of a case of intractable vomiting of pregnancy.

Second. Because of impossibility to apply Apostoli's treatment, as recommended in a former paper.

Third. As an excellent example of the importance of careful differentiation in selecting the proper form of current.

Fourth. Because of the prompt relief following the institution of the proper treatment.

On the 7th day of last Febuary I was summoned to attend the wife of Dr. H., whom I found very despondent, unable to retain anything, and suffering with constant nausea and very troublesome ptyalism. She vomited from fifty to sixty times in the twenty-four hours, and even when nothing had been ingested vomited quantities of sour liquid and mucus. The bowels were constipated. The conditions growing gradually worse; had been existing for three weeks, during which time various and numerous drugs and measures were tried, including teaspoonful doses of beer, and twenty-four hours trial of the Kelee chest postural treatment, without any benefit. It was then that the attending physicians referred Dr. H. to me for electrical treatment. On inquiry I found that the patient had been pregnant three times prior to this, and each time had been very sick with vomiting. Nausea and vomiting were present throughout the entire period of her first gestation, being especially severe during the first half. The labor was normal. In the early weeks of the second gestation, the same train of symptoms began, and increased in severity in spite of medication, until accidental abortion occurred some five weeks later.

The patient having lost her only child, and, being desirous of another, became pregnant a third time. When the third gestation had advanced to about six weeks, the nausea and vomiting made their appearance and fifteen days later the nausea had become constant and vomiting almost incessant. She could retain nothing and the salivary secretion was profuse and very troublesome. At first medication was given per orem, then rectal medication and alimentation had to be restorted to. Local treatment to the cervix, in the form of a mild dilatation and topical applications, and postural treatment were also tried and failed. Her condition growing daily more alarming, a consultation was held and it was decided to induce abortion in order to save her life. All the symptoms immediately disappeared after emptying the uterus, and she made an uneventful recovery.

With this history I appreciated at once that I had to deal with a case that would tax to the utmost the resources of electro-therapeutics. I instituted the treatment by trying to break the vicious cycle by Apostoli's method. This consists in positive galvanization of the pneumogastrics in their most accessible points in the neck.

The current is turned on gradually by means of a rheostat until all nausea disappears, and as soon as the patient experiences the nausea prodromal of the impending vomiting, the current is immediately and rapidly increased until either one of two things occurs—the nausea disappears or the patient complains of burning. At the end of about one minute the current is turned off, and then turned on again to the point at which it was at first.

During this treatment food or drink should be ingested and the application continued until all nausea and vomiting disappears, and the patient feels well and does not think she will vomit again. In this case it was impossible to apply the above technique because the vomiting was projectile in character and without prodromal nausea. I turned on the current until all nausea ceased, and at one moment the patient felt relieved and comfortable, and the next minute she was vomiting. After the ineffectual attempt to break the cycle, according to the above technique, I decided to employ a current strength of 10 to 15 m. a., with the positive pole over the vagus for twenty minutes, and then over the back of the neck for twenty minutes more, placing the negative pole over the epigastrium in both instances. This treatment I applied three times daily for four days with no result, other than a lessening of the nervous irritability, as evidenced by the disappearance of nervous cough and retching. On the sixth day I discontinued the use of galvanism and began that of Faradism, making the applications over the vagus in the neck, and over the back of the neck for brief periods, averaging five minutes in each situation. The improvement began at once and the patient was convalescent in forty-eight hours. I gave Dr. H. the necessary instructions and advised to continue the application twice daily for one week, thence once daily for another week. However, as Mrs. H. felt well, eating anything she desired, my advice was not heeded, and ten days later I was summoned again to find her relapsed in exactly the same condition as upon my first visit, but this time more nervous and despondent, and having lost all faith in the curative powers of electricity. Being aware that there was in this case a strong hereditary neurotic tendency, I immediately placed her under the treatment recommended for hysterical vomiting. This consisted in prolonged applications of a Faradic current of high tension for sedative effects.

Five days of this treatment, administering three times a day, gave negative results, and even seemed to aggravate the gastric and salivary secretions. The technique was then changed and brief applications of the same current for stimulating effects was used. The patient responded to the treatment at once, as she had done the first time, and was soon convalescent. This time my directions were strictly followed, and Dr. H., whom I saw again three days ago, told me that his wife was well and hearty; that the constipation was cured, the ptyalism disappeared, and she only vomited occasionally, after some emotion or fit of blues.

The most interesting question arising in the study of this case is: Why did the disease respond to this particular electric modality?

Before answering this question, let us consider the important role which the sympathetic system must necessarily play in pathological states of the viscera, and, in fact, in all reflex phenomena. This system controls, through the vaso-motor, the circulation, and hence the nutrition and functional activity of the various parts of the human organism. The action of all counter-irritant medication, by which deep-seated structures or organs are affected reflexedly, depends upon the influence on the sympathies of the agents employed. In medicine we use heat externally to cause relaxation and vaso-motor dilation in underlying structures, and cold to cause vaso-motor contraction. In electro-therapeutics we effect local sedation and reflex vaso-motor dilation by using prolonged applications of Faradic currents of high tension and local stimulation and reflex vaso-motor contraction by the brief application of the same currents.

These currents from a high-tension Faradic apparatus have a more decided influence on the vaso-motor than any other known therapeutic agent.

In the case of our patient the nausea, constipation, large amount of gastric mucus and salivary secretion indicated a condition of congestion and loss of tone, which were promptly cured by the tonic and vaso-motor contraction effects of the electric modality employed.

In the management of any case there are two facts which we should always bear in mind, viz.:

First. That after employing an electric current for a reasonable length of time, shorter in acute conditions, longer in subacute or chronic cases, without benefit, not to persist but make up mind that we are using the improper current.

Second. That when our results are negative and we are certain that our selection is correct according to the indication present, and our technique faultless, to give due consideration to personal equation.

We have idiosyncracies in electro-therapeutics, same as in medicinal therapeutics, and it not rarely happens that of two patients presenting the same conditions, one reacts best to the use of one form of electricity and the other to another. It follows, therefore, that to use electricity with satisfactory results we must have available the currents. I mention this fact specially because there are fads in medicine, and there is a tendency to employ newer apparatus which, according to the claims of the manufacturers, are superior to and can replace all the other currents; and the physician making use of one current to the exclusion of all others is certain to meet with failure and disappointment.

In conclusion, if I have succeeded in arousing your interest in this much-neglected branch, and in convincing you that electro-therapeutics is rational and scientific therapeutics, and not the empirical employment of a panacea of unknown characteristics, I will be fully repaid for the preparation of this paper.

